# Selection of outcome measurement instruments for a core outcome set for trials aimed at improving appropriate polypharmacy in older people in primary care: a Delphi consensus study

**DOI:** 10.1007/s11096-024-01780-4

**Published:** 2024-07-23

**Authors:** Mubarak N. Alqahtani, Heather E. Barry, Carmel M. Hughes

**Affiliations:** Primary Care Research Group, School of Pharmacy, Queen’s University Belfast, 97 Lisburn Road, Belfast, BT9 7BL UK

**Keywords:** COSMIN, Delphi questionnaire, Older people, Polypharmacy, Primary care

## Abstract

**Background:**

Despite developing a polypharmacy core outcome set (COS) in primary care, it is not clear how these outcomes should be measured.

**Aim:**

To select outcome measurement instruments (OMIs) for a COS targeting appropriate polypharmacy in older patients in primary care.

**Method:**

Following the Consensus-based Standards for the selection of health Measurement Instruments (COSMIN) guideline, OMIs were identified from a Cochrane review focusing on appropriate polypharmacy. The quality of OMIs was assessed using a published checklist. Subsequently, two rounds of Delphi questionnaires were conducted via the SoGoSurvey^®^ platform, engaging stakeholders (researchers, clinicians and journal editors specialising in geriatric primary care) to achieve consensus on OMIs using a scale encompassing “agree”, “disagree”, or “unsure”. Consensus was achieved if 70% or more participants chose “agree” and 15% or fewer chose “disagree.”

**Results:**

The quality of 20 OMIs identified from the Cochrane review was evaluated. Seven OMIs were selected based on meeting the COSMIN guideline’s minimum requirements. Out of 188 potential participants, 57 (30.3%) consented to participate. Rounds 1 and 2 of Delphi exercises were completed by 50 respondents, achieving agreement on three OMIs: ‘number of serious adverse drug reactions (ADRs)’ (98%), ‘number of deaths’ (76%), and ‘number of patients who fell’ (70%) for measuring ‘serious ADRs,’ ‘mortality,’ and ‘falls,’ respectively. No agreement was reached for ‘medication appropriateness,’ ‘medication side-effects,’ ‘quality of life,’ and ‘medication regimen complexity.’

**Conclusion:**

OMIs were selected for a limited number of outcomes in the polypharmacy COS. Future research should identify suitable OMIs for the remaining four outcomes.

**Supplementary Information:**

The online version contains supplementary material available at 10.1007/s11096-024-01780-4.

## Impact statements

- The research demonstrates the practical applicability of following the Consensus-based Standards for the selection of health Measurement Instruments (COSMIN) initiative’s guidance for selecting outcome measurement instruments (OMIs) for a core outcome set (COS) focusing on polypharmacy in older adults in primary care.
- The three identified OMIs, ‘number of serious ADRs,’ ‘number of deaths,’ and ‘number of patients who fell’ provide methods to evaluate the impact of polypharmacy interventions on older people in primary care.
- Using these OMIs, together with the polypharmacy COS in primary care settings, can help in measuring and comparing the effects of interventions that are tailored towards improving appropriate polypharmacy.
- These OMIs could be directly applied in clinical practice to monitor and improve patient outcomes, thereby improving the safety and efficacy of polypharmacy management

## Introduction

Polypharmacy is increasingly recognised as beneficial, especially for managing multimorbidity [1, 2]. However, balancing appropriate and inappropriate polypharmacy remains challenging [3]. Inconsistent reporting in polypharmacy studies complicates drawing conclusions about intervention effectiveness [4]. To address this, the Core Outcome Measures for Effectiveness Trials (COMET) Initiative promotes developing core outcome sets (COS) [5]. A COS comprises standardised, agreed-upon outcomes important to measure and report as a minimum in all trials within a particular health area [6]. A COS for evaluating appropriate polypharmacy interventions in older people in primary care has been developed [4], aiding in trial comparisons and identifying effective interventions.

Despite the development of a number of COSs, little research has focused on selecting Outcome Measurement Instruments (OMIs) for studies [5]. An OMI represents a tool or approach that dictates *‘how to’* measure a specific construct in terms of quality and quantity [7]. Multiple instruments for the same outcome can lead to variations and data synthesis challenges in systematic reviews [8, 9]. The COMET and Consensus-based Standards for the selection of health Measurement Instruments (COSMIN) initiatives aim to establish standards for selecting OMIs for COS outcomes by identifying existing OMIs from systematic reviews or other sources, evaluating their quality, selecting one OMI per outcome, and using stakeholder consensus [7]. Although a COS exists for trials targeting appropriate polypharmacy in older adults in primary care [4], no research has determined which OMIs to use with it.

### Aim

This study aimed to reach consensus on OMIs for outcomes included in the polypharmacy COS for trials targeting appropriate polypharmacy in primary care for older patients.

### Ethics approval

The study was approved by Ethics Committee of the Queen’s University Belfast (Ref: MHLS21_85; date of approval: July 28, 2021) in accordance with the Declaration of Helsinki. All participants provided written informed consent.

## Method

**Fig. 1:**
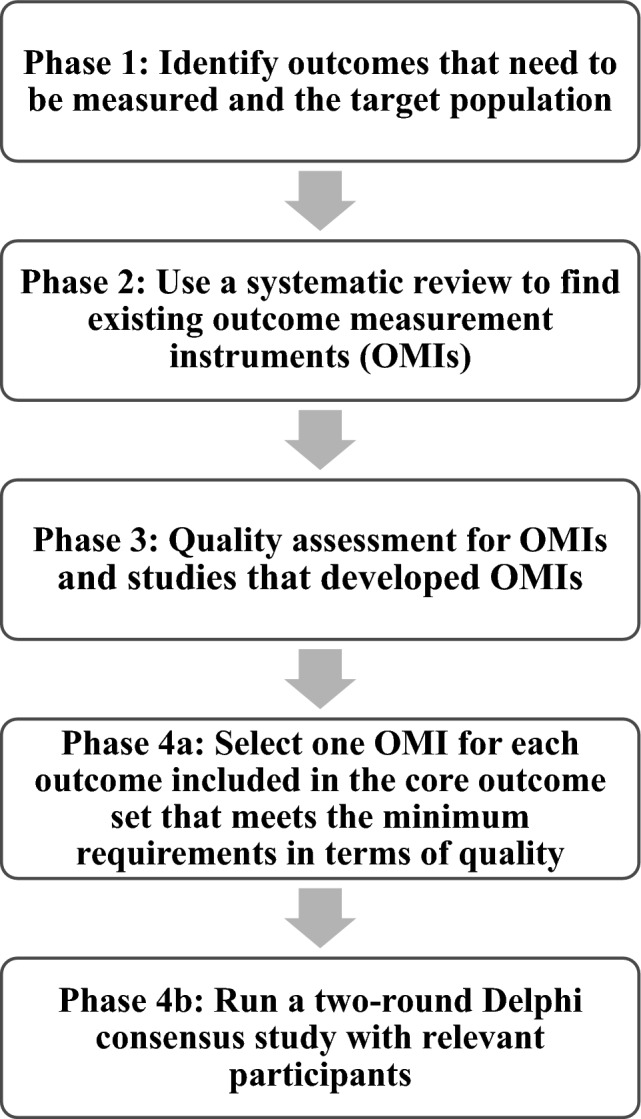
Process of selecting outcome measurement instruments for a core outcome set

This study adhered to the COSMIN Initiative’s for selecting OMIs for a COS which encompasses four phases (Fig. 1).

### Phase 1: Identifying outcomes for the OMIs

The target population was older patients (≥ 65 years) in primary care. We identified outcomes from the previously developed polypharmacy COS [4]. Among 16 outcomes in the COS, the seven highest ranking outcomes were prioritised based on published guidance [4]. Measuring more than seven outcomes could be impractical [10]. The seven highest ranking outcomes in the COS in primary care were serious adverse drug reactions (ADRs), medication appropriateness, falls, medication regimen complexity, quality of life (QoL), mortality, and medication side effects.

### Phase 2: Finding existing OMIs

Systematic reviews are recommended by COSMIN guidance to identify existing OMIs [7]. This study extracted OMIs from randomised clinical trials (RCTs) included in our Cochrane review [11], which focused on interventions targeting appropriate polypharmacy in primary care.

### Phase 3: Quality assessment of OMIs

The COSMIN checklist was used to evaluate the methodological quality of studies that developed OMIs relevant to the COS [7]. OMIs identified from the Cochrane review were categorised into ‘objective’ or ‘subjective’ based on their nature. Objective OMIs were evaluated for both content validity and feasibility aspects, whereas subjective OMIs were assessed for content validity, feasibility aspects and if applicable, other measurement properties. Two researchers independently evaluated the quality of OMIs, with discrepancies reviewed by a third researcher. The overall quality of OMIs was rated depending on the results of the assessments of their measurement properties and if applicable, their feasibility aspects [7].

### Phase 4a: Selection of OMIs for outcomes included in the COS

Following the quality evaluation in Phase 3, one OMI was chosen for every outcome in the polypharmacy COS that fulfilled the best minimum requirements for selection and which was feasible to use [7, 8].

### Phase 4b: Consensus procedure for OMIs

The Delphi approach was employed to achieve consensus on OMIs to use with the COS. The Delphi approach involves distributing a questionnaire to key stakeholders consisting of a series of items which are scored over a number of sequential rounds to reach consensus [12, 13].

### Delphi questionnaire development

For the online Delphi exercise, the research team created an electronic questionnaire. This comprised a list of OMIs for the polypharmacy COS based on work undertaken in Phases 1–3, together with their definitions and illustrative examples (Supplementary Figure S1). Two versions of the questionnaire, one for researchers/academics and one for the public, were created and distributed using the SoGoSurvey^®^ platform. Before distribution, the questionnaires were piloted with twelve members of the Primary Care Research Group at the School of Pharmacy, Queen’s University Belfast, and no modifications were required. Estimated completion time was 10 min.

### Recruitment of Delphi panel members

Eligible participants included researchers or clinicians specialising in the care or prescribing for older adults in primary care, journal editors in geriatric medicine, or representatives of older patient interests (such as charities). There are no guidelines for the ideal Delphi panel size, with previous studies ranging from 12 to 311 participants [5]. Previous Delphi studies typically achieved a 30–40% recruitment rate [14, 15]. Expecting 45–60 participants (30–40%), we aimed to invite at least 150 potential participants to achieve this rate. International recruitment efforts were made to ensure the selection of OMIs had international relevance.

Based on the study team’s knowledge of primary healthcare, researchers’ publication records, and professional activities, a list of potential participants was compiled. The study team also contacted the Patient and Client Council, which represents the interests of people who use health and social care services in Northern Ireland, to recruit participants from this group. Invitation emails were sent to potential participants, who were asked to complete a consent form, which was included in the first questionnaire.

### Delphi process

To reach consensus on OMIs for the polypharmacy COS, two rounds of the two versions of the Delphi surveys were distributed via email to participants.

### Delphi Round 1 (21st June 2022–5th July 2022)

Participants provided demographic information at the start of the survey. Stakeholders indicated their agreement with using the selected OMI to measure the associated outcome by choosing from ‘agree’, ‘disagree’, or ‘unsure’. If ‘unsure’ or ‘disagree’ was chosen, a text box prompted the participant to provide a brief explanation. Participants were encouraged to suggest other OMIs for outcomes not presented in Round 1.

Following Round 1, participants’ response rate to the survey and the percentage of respondents who selected ‘agree’, ‘disagree’ and ‘unsure’ for OMIs were calculated. Any additional OMIs reported by participants were checked to ensure there was no overlap with OMIs previously presented in Round 1.

### Delphi Round 2 (11th July 2022–14th August 2022)

Round 2 proceeded in the same manner as Round 1. Every participant received their Round 1 responses for each OMI along with the Round 2 email. Additionally, the Round 2 questionnaire included a summary of all Round 1 respondents' collective responses for each OMI to enable comparison. All OMIs from Round 1 were included in Round 2. The response rate and distribution of scores were determined similarly as in Round 1.

### Data analysis

The Delphi questionnaire employed three scoring categories (agree, disagree, and unsure), a method previously used by Delphi questionnaires in the selection of OMIs for COSs [14–16]. Consensus for inclusion of an OMI to measure the polypharmacy COS was achieved if at least 70% of the respondents selected ‘agree’ and 15% or less selected ‘disagree’. Thereafter, OMIs were classified as ‘consensus in’ or ‘no consensus’. If an OMI was deemed ‘consensus in,’ it was included in the list of OMIs for the polypharmacy COS.

## Results

In Phase 1, the seven highest ranking outcomes in the polypharmacy COS were identified: medication appropriateness, serious ADRs, QoL, medication regimen complexity, falls, medication side-effects and mortality [4].

In Phase 2, OMIs were extracted from trials conducted in primary care that were included in the Cochrane review [11]. Thirty-six studies were involved in the evaluation of OMI quality. The research team compiled a list of these OMIs, which included 20 OMIs to measure outcomes pertaining to the outcomes in the polypharmacy COS (Table 1).

**Table 1:** A list of identified and selected outcome measurement instruments for the polypharmacy core outcome set [11]

Outcome	Identified OMI	Selected OMIs for each outcome that were presented to the Delphi panel^a^
Serious ADRs	Number of ADRs	Number of ADRs
Medication appropriateness	Screening Tool of Older Person’s Prescriptions/Screening Tool to Alert to Right Treatment (STOPP/START)	MAI
	Medication Appropriateness Index (MAI)	
	MAI (modified, excluded cost-effectiveness from MAI items)	
	PRISCUS criteria	
	McLeod criteria	
	The Tool to Reduce Inappropriate Medication (TRIM)	
	The Swedish Criteria drug-specific quality indicators established by the Swedish National Board of Health and Welfare	
	Beers Criteria	
Falls	Number of patients who fell	Number of patients who fell
Medication regimen complexity	Total number of prescriptions	Total number of prescriptions
	Number of single doses/day	
	Medication Regimen Complexity Index (MRCI)	
Quality of life	EuroQol five dimensions (EQ-5D) index	EQ-5D
	The Quality of Life in Alzheimer’s Disease instrument (QoL-AD)	
	The Medical Outcomes Study 36-Item Short-Form Health Survey (SF-36)	
	12-Item Short Form Survey (SF-12)	
	The 15-dimensional instrument of health-related quality of life (15D)	
Mortality	Number of deaths (number of people who have died)	Number of deaths
Medication side-effects	Number of symptoms of side-effects	Number of symptoms of side-effects

^a^The selection of OMIs based on evaluation of the quality of OMIs process that followed COSMIN guideline

In Phase 3, the COSMIN checklist was used to assess the methodological quality of studies that developed the OMIs as the first step in the quality evaluation of the instruments [7]. Overall quality was considered “doubtful” for one study, while 22 studies were considered “inadequate” in terms of overall quality (Supplementary Table S1). Subsequently, OMIs were then evaluated and categorised as either “objective” or “subjective” OMIs. Following the COSMIN guidance for evaluating the quality of OMIs, the quality of all objective OMIs was deemed to be high (Supplementary Table S2). In contrast, all subjective OMIs were classified as low to very low in terms of quality (Supplementary Table S3).

In Phase 4, the research team chose one OMI for every outcome based on the results of the quality evaluation (Table 1). This selection was in accordance with which instrument best met the COSMIN guideline minimum requirements to include an OMI to measure an outcome: good content validity, being feasible to use, and having the highest rating for other measurement properties [7]. The seven selected OMIs were included in the Delphi questionnaire.

### Demographics of participants

Initially, 188 emails inviting potential participants to take part were distributed. A total of 57 potential participants (30.3% recruitment rate) consented to participate in the study. Fifty participants completed the questionnaires [n = 28 (56%) males and n = 22 (44%) females]. The median age was 51 years. The majority of respondents (n = 27; 54%) lived in Europe (outside the UK), followed by the UK (n = 12; 24%), North America (n = 6; 12%), Oceania (n = 4; 8%) and Asia (n = 1; 2%). The majority of respondents were classified as researchers. None of the questionnaires was completed by members of the public (Table 2).

**Table 2:** Demographic details of respondents to the Delphi questionnaires

Characteristics	Round 1 and 2
Participant, n	50
Gender, n (%)	
Male	28 (56%)
Female	22 (44%)
Age (years), median (range)	51 (31–78)
Continent of residence, n (%)	
UK	12 (24%)
Europe (outside the UK)	27 (54%)
North America	6 (12%)
Asia	1 (2%)
Oceania	4 (8%)
Professional area, n (%)^a^	
Doctor	17 (34%)
Pharmacist	29 (58%)
Researcher/academic	36 (72%)
Journal editor	8 (16%)
Healthcare staff member/ manager	7 (14%)
Public participants	0 (0%)
Other	3 (6%)

^a^Because multiple responses from each participant were possible, the total percentage may exceed 100%

### Round 1 questionnaire

Fifty of the 57 participants who agreed to participate in the study completed Round 1 (response rate: 87.7%). Following completion of this round, 90% (n = 45) of the participants were in agreement that the OMI ‘number of serious ADRs’ should be used to measure ‘serious ADRs’ and consensus was reached for this OMI. No other OMIs in Round 1 achieved consensus (Table 3). Participant comments provided in Round 1 responses were checked. No additional OMIs were added to Round 2 as those suggested by respondents in Round 1 did not differ from those already included.

**Table 3:** Round 1 distribution of consensus level of participants for each OMI

Outcome and measurement instrument (n = 7)	Participants’ level of agreement (n = 50)		
	Agree n (%)	Disagree n (%)	Unsure n (%)
*Outcome:* Serious adverse drug reactions *OMI:* The number of serious adverse drug reactions	45 (90%)	2 (4%)	3 (6%)
*Outcome:* Mortality *OMI:* The number of deaths	32 (64%)	6 (12%)	12 (24%)
*Outcome:* Falls *OMI:* The number of patients who fell	29 (58%)	6 (12%)	15 (30%)
*Outcome:* Medication appropriateness *OMI*: Medication appropriateness index	25 (50%)	13 (26%)	12 (24%)
*Outcome:* Medication side-effects *OMI:* The number of symptoms of side-effects	25 (50%)	7 (14%)	18 (36%)
*Outcome:* Quality of life *OMI:* EQ-5D	34 (68%)	7 (14%)	9 (18%)
*Outcome:* Medication regimen complexity *OMI:* The total number of prescriptions	27 (54%)	6 (12%)	17 (34%)

*OMI* outcome measurement instrument

### Round 2 questionnaire

The Round 2 questionnaire was distributed to only those who had completed Round 1 (n = 50). It was completed by all respondents who took part in Round 1 (response rate: 100%). Following Round 2, the percentage of agreement had increased from Round 1 for the OMI ‘number of serious ADRs’ (98%) to measure ‘serious ADRs’. Two additional OMIs—‘number of deaths’ (76%) and ‘number of patients who fell’ (70%) to measure ‘mortality’ and ‘falls’ respectively, also reached consensus (Table 4). There was no agreement on the remaining OMIs to measure “medication appropriateness,” “medication side-effects,” “QoL,” and “medication regimen complexity”. Therefore, these outcomes were stated as ‘OMI not available’.

**Table 4:** Round 2 distribution of consensus level of participants for each OMI

Outcome and measurement instrument (n = 7)	Participants’ level of agreement (n = 50)		
	Agree n (%)	Disagree n (%)	Unsure n (%)
*Outcome:* Serious adverse drug reactions *OMI:* The number of serious adverse drug reactions	49 (98%)	0 (0%)	1 (2%)
*Outcome:* Mortality *OMI:* The number of deaths	38 (76%)	6 (12%)	6 (12%)
*Outcome:* Falls *OMI:* The number of patients who fell	35 (70%)	5 (10%)	10 (20%)
*Outcome:* Medication appropriateness *OMI*: Medication appropriateness index	32 (64%)	5 (10%)	13 (26%)
*Outcome:* Medication side-effects *OMI:* The number of symptoms of side-effects	30 (60%)	7 (14%)	13 (26%)
*Outcome:* Quality of life *OMI:* EQ-5D	28 (56%)	11 (22%)	11 (22%)
*Outcome:* Medication regimen complexity *OMI:* The total number of prescriptions	26 (52%)	15 (30%)	9 (18%)

*OMI* outcome measurement instrument

## Discussion

### Statement of key findings

The COSMIN Initiative’s guidelines for selecting OMIs for a COS were utilised to choose OMIs for the polypharmacy COS [7]. Using the COSMIN checklist, 36 studies were evaluated for OMI quality, with most being judged as ‘inadequate’. According to the COSMIN guideline, the lowest rating of any standard in the COSMIN checklist is utilised to determine the overall study’s quality [7]. Subsequently, 20 OMIs related to the polypharmacy COS were identified and evaluated for quality. All OMIs for measuring ‘serious ADRs’, ‘falls’, ‘mortality’, and ‘medication side-effects’ were categorised as ‘objective’. For ‘medication regimen complexity’, three OMIs were identified, with two (‘total number of prescriptions’ and ‘number of single doses/day’) classified as ‘objective’ and one [‘Medication Regimen Complexity Index (MRCI)’] as ‘subjective’ [7]. All OMIs used to measure ‘QoL’ and ‘medication appropriateness’ were considered ‘subjective’. Objective OMIs were assessed for content validity and feasibility aspects, while subjective OMIs were assessed for content validity, feasibility aspects and other measurement properties (where applicable). Overall, all objective OMIs were assessed as being high in quality, whereas subjective OMIs was deemed low or very low. This may be attributed to subjective OMIs being dependent on assessors’ judgment. In contrast, objective OMIs were independent from assessors’ judgment and calculated as quantitative data.

### Strengths and weaknesses

This research has several strengths. It is the first study to select OMIs for outcomes included in the polypharmacy COS by adhering to COMET and COSMIN guidelines. Every OMI was extracted from RCTs included in a Cochrane review [11]. The consensus process involved a Delphi panel from various countries and professional backgrounds. The response rates were high for both Delphi rounds.

There are a number of limitations. Despite achieving the expected number of respondents (n = 50), a low recruitment rate of 30.3% was recorded. COVID-19 restrictions prevented a recommended plenary discussion and voting during a face-to-face meeting for consensus taking place, prompting the use of an online Delphi procedure, which has been successful in previous studies [4, 15]. Efforts to involve public representatives faced challenges due to the perceived complexity of the study, potentially hindering recruitment from advocacy groups for older people.

### Interpretation of findings

Despite their common use, OMIs for measuring ‘medication appropriateness’ and ‘QoL’ outcomes did not meet COSMIN criteria for good measurement properties. However, the MAI and EQ-5D were the most highly rated in terms of quality evaluation amongst all OMIs for measuring ‘medication appropriateness’ and ‘QoL’, respectively. Hence, they were included in the Delphi survey as the associated outcomes ‘medication appropriateness’ and ‘QoL’ had been highly ranked in the polypharmacy COS [4]. Three OMIs measured medication regimen complexity, with the MRCI deemed subjective and excluded from the polypharmacy COS as it did not meet the COSMIN criteria for good measurement properties. The other two OMIs, ‘total number of prescriptions’ and ‘number of single doses/day’, were classified as objective and met COSMIN criteria. To select one suitable OMI for measuring medication regimen complexity from these two OMIs, ‘total number of prescriptions’ was chosen based on evaluation of feasibility.

The seven OMIs that were supported by the best quality assessments were selected for the Delphi exercise. Fifty participants completed the Delphi surveys via two rounds. In the Delphi questionnaire, the recruitment rate was low (30.3%), however, it was consistent with similar Delphi studies (30–40%) [14, 15]. This study recorded high response rates (Round 1: 87.7%; Round 2: 100%), exceeding those of previous studies (Round 1: 44%; Round 2: 41%) [14], (Round 1: 86.8%; Round 2: 91.5%) [15], and ranging from 40 to 60% in a four-round study [17]. Public participants declined participation, which has also been observed in previous studies as well [14, 15].

Following the Delphi exercise, consensus was reached on three OMIs to measure three outcomes included in the polypharmacy COS, namely ‘number of serious ADRs’, ‘number of deaths’ and ‘number of patients who fell’. All these selected OMIs were objective, i.e. independent from assessor judgments, quantitative and easy to understand [18, 19]. No consensus was reached for other OMIs.

Despite no OMIs being selected for ‘medication appropriateness’, ‘QoL’, ‘medication regimen complexity’ and ‘medication side-effects’ in this study, previous studies used OMIs that are potentially applicable to the polypharmacy COS. For example, the Assessment of Underutilization of Medication (AOU) tool [20], Assessing Care of Vulnerable Elderly (ACOVE) indicators [21], the Meds75 + database [22], the Rationalization of Home Medication by an Adjusted STOPP in Older Patients (RASP) list [23] and Fit fOR The Aged (FORTA) criteria [24] used in literature to measure ‘medication appropriateness’. A study reported that a combination of implicit and explicit tools can measure medication appropriateness [25]. However, they were excluded from this study as these tools were not utilised in primary care-based trials included in the Cochrane review [11]. Similarly, a range of tools used in studies to measure QoL, for instance, medication-related QoL (MRQoL) [26, 27], the QUALIDEM [28] and ICECAP-O [29]. Again, these latter instruments were not included in this study as they were not used in primary care-based studies. For ‘medication regimen complexity’, five tools were reported in a systematic review as reliable for measuring medication regimen complexity [30]. They were: the MRCI [31], the Medication Complexity Index (MCI) [32], the Epilepsy Medication Treatment Complexity Index (EMTCI) [33], Antiretroviral Regimen Complexity (ARC) [34] and the Antiretroviral Medication Complexity Index (AMCI) [35]. The MRCI, however, was excluded in this present research arising from methodological and quality issues; for example, the study that developed the MRCI had a sample size lower than 30 [31], which is interpreted by the COSMIN checklist as inadequate [7]. The other four tools could measure medication regimen complexity, although some are disease-specific instruments, for instance, the AMCI and ARC are used for HIV, and the EMTCI scale is used for epilepsy. As noted above, ‘number of single doses/day’ tool met the COSMIN criteria for good measurement properties, thus, it could be used to measure medication regimen complexity. Regarding ‘medication side-effects’, questionnaires such as the Patient Reported Adverse Drug Event Questionnaire [36] and the Maudsley Side-Effects (MSE) tool [37] were used in previous studies to measure medication side-effects in older people. However, the reliability and validity of these tools require testing to identify the suitable one that can measure this outcome in older people in primary care.

### Further research

Consensus was reached for the OMIs ‘number of serious ADRs’, ‘number of deaths’, and ‘number of patients who fell’ for measuring ‘serious ADRs’, ‘mortality’, and ‘falls’, respectively. These three selected OMIs can be used in future studies to measure and compare the effects of interventions targeting the appropriateness of polypharmacy in older patients.

As no consensus was reached for OMIs to measure ‘medication appropriateness’, ‘medication side effects’, ‘QoL’ and ‘medication regimen complexity’, further research may develop new OMIs for these four remaining outcomes in the polypharmacy COS. Future studies may also examine and use such OMIs that used in other health settings (hospital, care home) for measuring these four remaining outcomes.

## Conclusion

The research has culminated in agreement on OMIs for some outcomes in the polypharmacy COS recommended for use in trials targeting appropriate polypharmacy. Following the COSMIN guidelines, ‘number of serious ADRs’, ‘number of deaths’ and ‘number of patients who fell’ OMIs were chosen for measuring ‘serious ADRs’, ‘mortality’ and ‘falls’, respectively.

## Supplementary Information

Below is the link to the electronic supplementary material.

Supplementary file 1 (DOCX 200 kb)
