# Supplementary material for: Selection of outcome measurement instruments for a core outcome set for trials aimed at improving appropriate polypharmacy in older people in primary care: a Delphi consensus study
Source: Int J Clin Pharm. 2024 Jul 23;46(6):1391–9. doi: 10.1007/s11096-024-01780-4 (PMC11576772; doi:10.1007/s11096-024-01780-4)
Supplement: Supplementary file 1 — (DOCX 200 kb) [file 11096_2024_1780_MOESM1_ESM.docx]

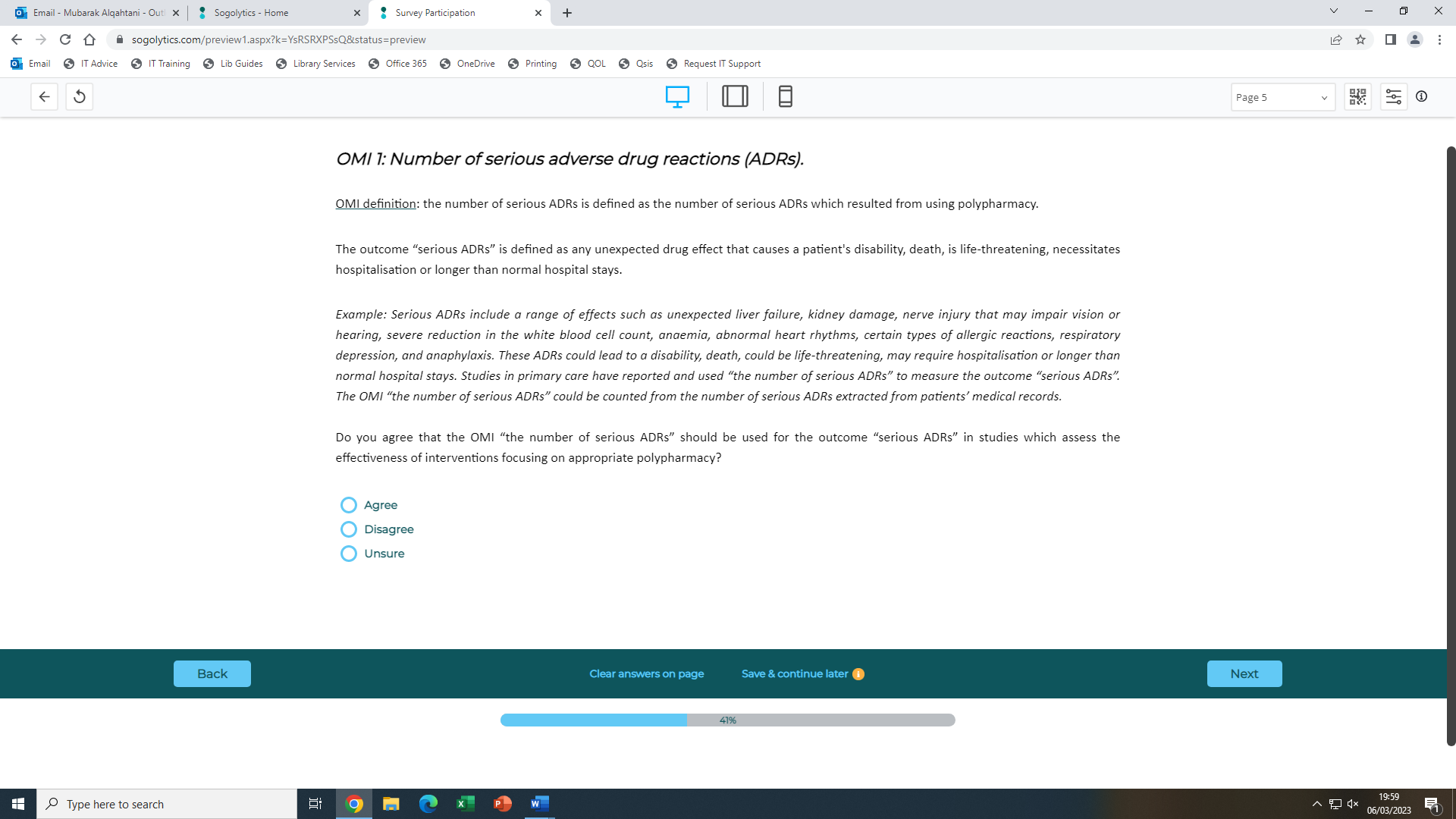


**Figure S1** A screenshot of a question included in the Delphi questionnaire.

**Table S1** Evaluation of the methodological quality of studies which developed OMIs to measure outcomes included in the COS

| **OMI** | **Outcome** | **Study** | **Methodological** **quality of developmental studies** |
| --- | --- | --- | --- |
| STOPP/START | Medication appropriateness | Gallagher et al. [1] | Inadequate |
|  |  | O’Mahony et al. [2] | Inadequate |
| MAI | Medication appropriateness | Hanlon et al. [3] | Inadequate |
|  |  | Samsa et al. [4] | Inadequate |
| PRISCUS criteria | Medication appropriateness | Holt et al. [5] | Inadequate |
| McLeod criteria | Medication appropriateness | McLeod et al. [6] | Inadequate |
| TRIM | Medication appropriateness | Niehoff et al. [7] | Inadequate |
|  |  | Fried et al. [8] | Inadequate |
| The Swedish Criteria | Medication appropriateness | Fastbom et al. [9] | Inadequate |
|  |  | Fastbom and Schmidt. [10] | Inadequate |
|  |  | Fastbom et al. [11] | Inadequate |
| Beers Criteria | Medication appropriateness | Beers et al. [12] | Inadequate |
|  |  | Beers [13] | Inadequate |
|  |  | Fick et al. [14] | Inadequate |
|  |  | AGS [15] | Inadequate |
|  |  | AGS [16] | Inadequate |
|  |  | AGS [17] | Inadequate |
| MRCI | Medication Regimen Complexity | George et al. [18] | Inadequate |
| EQ-5D index | Quality of life | EuroQol Group [19] | Doubtful |
| QoL-AD | Quality of life | Logsdon et al. [20] | Inadequate |
|  |  | Logsdon et al. [21] | Inadequate |
| SF-36 | Quality of life | Ware and Sherbourne [22] | Inadequate |
|  |  | Ware [23] | Inadequate |
| SF-12 | Quality of life | Ware et al. [24] | Inadequate |
| 15D | Quality of life | Sintonen and Pekurinen [25] | Inadequate |
|  |  | Sintonen [26] | Inadequate |

**Table S2** Summary of quality assessment for ‘objective’ outcome measurement instruments.

| **Outcome measurement instrument** | **Content validity** | **Feasibility aspects** | **Overall quality of evidence** |
| --- | --- | --- | --- |
| **Outcome: Falls** | | | |
| The number of patients who fell [27] | **+** | **+** | **+** |
| **Outcome: Serious adverse drug reactions (ADRs)** | | | |
| Number of ADRs [27,28,29,30,31] | **+** | **+** | **+** |
| **Outcome: Mortality** | | | |
| The number of deaths [27,32] | **+** | **+** | **+** |
| **Outcome: Medication regimen complexity** | | | |
| Total number of prescriptions [27,28] | **+** | **+** | **+** |
| Number of single doses/day [27,28] | **+** | **+** | **+** |
| **Outcome: Medication side-effects** | | | |
| Number of symptoms of side-effects [27,28] | **+** | **+** | **+** |

**(+) =** positive rating.

**Table S3** Summary of quality assessment for ‘subjective’ outcome measurement instruments

| **Outcome measurement instrument** | **Content validity** | **Structural validity** | **Internal consistency** | **Cross‐cultural validity** | **Reliability** | **Measurement error** | **Criterion validity** | **Hypotheses testing** | **Responsiveness** | **feasibility**  **aspects** | **Overall quality of evidence** |
| --- | --- | --- | --- | --- | --- | --- | --- | --- | --- | --- | --- |
| Outcome: Medication appropriateness | | | | | | | | | | | |
| STOPP/START [1,2]. | ? | NA | NA | + | NA | ? | - | NA | NA | ? | Very low |
| MAI [3,4]. | ? | NA | NA | - | + | + | - | NA | - | + | Very low |
| PRISCUS criteria [5]. | ? | NA | NA | - | - | ? | - | ? | - | ? | Very low |
| McLeod criteria [6]. | ? | NA | NA | - | - | ? | - | - | - | ? | Very low |
| TRIM [7,8] | ? | NA | - | - | - | NA | - | ? | - | ? | Very low |
| The Swedish Criteria drug-specific quality indicators established by the Swedish National Board of Health and Welfare [9,10,11]. | ? | NA | NA | - | ? | ? | NA | NA | NA | ? | Very low |
| Beers Criteria [12,13,14,15,16,17]. | ? | NA | NA | - | ? | ? | NA | NA | - | ? | Very low |

**Table S3 (*cont’d)*** Summary of quality assessment for ‘subjective’ outcome measurement instruments

| **Outcome measurement instrument** | **Content validity** | **Structural validity** | **Internal consistency** | **Cross‐cultural validity** | **Reliability** | **Measurement error** | **Criterion validity** | **Hypotheses testing** | **Responsiveness** | **feasibility**  **aspects** | **Overall quality of evidence** |
| --- | --- | --- | --- | --- | --- | --- | --- | --- | --- | --- | --- |
| Outcome: Quality of life | | | | | | | | | | | |
| EQ-5D [19]. | ? | NA | NA | + | + | + | NA | NA | NA | + | Low |
| QoL-AD [20,21]. | ? | NA | ? | - | - | ? | - | ? | - | ? | Very low |
| SF-36 [22,23]. | ? | NA | NA | - | NA | ? | - | NA | NA | ? | Very low |
| SF-12 [24]. | ? | NA | - | ? | - | NA | - | ? | - | + | Very low |
| 15D [25,26]. | ? | NA | NA | + | - | ? | - | ? | - | + | Very low |
| Outcome: Medication regimen complexity | | | | | | | | | | | |
| MRCI [18]. | ? | - | NA | + | - | ? | - | + | - | ? | Very low |

**(+)=**Positive rating, (?)=Indeterminate rating, (-)=Negative rating, NA=Not applicable.

**References**

1. Gallagher P, Ryan C, Byrne S et al. STOPP (Screening Tool of Older Person's Prescriptions) and START (Screening Tool to Alert doctors to Right Treatment). Consensus validation. *Int J Clin Pharmacol Ther*. 2008;46(2):72-83. <https://doi.org/10.5414/cpp46072>
2. O'Mahony D, O'Sullivan D, Byrne S et al. STOPP/START criteria for potentially inappropriate prescribing in older people: version 2. *Age Ageing*. 2015;44(2):213-218. <https://doi.org/10.1093/ageing/afu145>
3. Hanlon J, Schmader K, Samsa G et al. A method for assessing drug therapy appropriateness. *J Clin Epidemiol*. 1992;45(10):1045-1051. <https://doi.org/10.1016/0895-4356(92)90144-c>
4. Samsa G, Hanlon J, Schmader K et al. A summated score for the medication appropriateness index: development and assessment of clinimetric properties including content validity. *J Clin Epidemiol*. 1994;47(8):891-896. <https://doi.org/10.1016/0895-4356(94)90192-9>
5. Holt S, Schmiedl S, Thürmann PA. Potentially inappropriate medications in the elderly: the PRISCUS list. *Dtsch Arztebl Int*. 2010;107(31-32):543-551. <https://doi.org/10.3238/arztebl.2010.0543>
6. McLeod P, Huang A, Tamblyn R et al. Defining inappropriate practices in prescribing for elderly people: a national consensus panel. *CMAJ*. 1997;156(3):385-391.
7. Niehoff K, Rajeevan N, Charpentier P et al. Development of the Tool to Reduce Inappropriate Medications (TRIM): A Clinical Decision Support System to Improve Medication Prescribing for Older Adults. *Pharmacotherapy*. 2016;36(6):694-701. <https://doi.org/10.1002/phar.1751>
8. Fried T, Niehoff K, Street R et al. Effect of the Tool to Reduce Inappropriate Medications on Medication Communication and Deprescribing. *J Am Geriatr Soc*. 2017;65(10):2265-2271. <https://doi.org/10.1111/jgs.15042>
9. Fastbom J, Dani-elsson B, Wikner B. Indikatorer för god läkemedelsterapi hos äldre. The Swedish National Bord for Health and Welfare. 2017. <https://www.socialstyrelsen.se/globalassets/sharepoint-dokument/artikelkatalog/ovrigt/2017-6-7.pdf> Accessed 02.06.2022.
10. Fastbom J, Schmidt I, Lieberman-Ram H et al. Indikatorer för utvärdering av kvaliteten i äldres läkemedelsterapi. The Swedish National Bord for Health and Welfare S*ocialstyrelsen*. 2004:110-120.
11. Fastbom J, Schmidt I. Indikatorer för god läkemedelsterapi hos äldre. The Swedish National Bord for Health and Welfare. 2010. <https://www.socialstyrelsen.se/publikationer2010/2010-6-29>. Accessed: 02. 06. 2022.
12. Beers M, Ouslander J, Rollingher I et al. Explicit criteria for determining inappropriate medication use in nursing home residents. UCLA Division of Geriatric Medicine. *Arch Intern Med*. 1991;151(9):1825-1832.
13. Beers M. Explicit criteria for determining potentially inappropriate medication use by the elderly. An update. *Arch Intern Med*. 1997;157(14):1531-1536.
14. Fick D, Cooper J, Wade W et al. Updating the Beers criteria for potentially inappropriate medication use in older adults: results of a US consensus panel of experts [published correction appears in Arch Intern Med. *Arch Intern Med*. 2003;163(22):2716-2724. <https://doi.org/10.1001/archinte.163.22.2716>
15. American Geriatrics Society 2012 Beers Criteria Update Expert Panel. American Geriatrics Society updated Beers Criteria for potentially inappropriate medication use in older adults. *J Am Geriatr Soc*. 2012;60(4):616-631. <https://doi.org/10.1111/j.1532-5415.2012.03923.x>
16. American Geriatrics Society 2015 Beers Criteria Update Expert Panel. American Geriatrics Society 2015 Updated Beers Criteria for Potentially Inappropriate Medication Use in Older Adults. *J Am Geriatr Soc*. 2015;63(11):2227-2246. <https://doi.org/10.1111/jgs.13702>
17. American Geriatrics Society Beers Criteria® Update Expert Panel. American Geriatrics Society 2019 Updated AGS Beers Criteria® for Potentially Inappropriate Medication Use in Older Adults. *J Am Geriatr Soc*. 2019;67(4):674-694. <https://doi.org/10.1111/jgs.15767>
18. George J, Phun Y, Bailey M et al. Development and validation of the medication regimen complexity index. *Ann Pharmacother*. 2004;38(9):1369-1376. <https://doi.org/10.1345/aph.1D479>
19. EuroQol Group. EuroQol--a new facility for the measurement of health-related quality of life. *Health Policy*. 1990;16(3):199-208. <https://doi.org/10.1016/0168-8510(90)90421-9>
20. Logsdon R, Gibbons L, McCurry S et al. Quality of life in Alzheimer's disease: patient and caregiver reports. *Journal of Mental Health and Aging*. 1999; 5:21-32.
21. Logsdon R, Gibbons L, McCurry S et al. Assessing quality of life in older adults with cognitive impairment. *Psychosom Med*. 2002;64(3):510-519. <https://doi.org/10.1097/00006842-200205000-00016>
22. Ware J, Sherbourne C. The MOS 36-item short-form health survey (SF-36). I. Conceptual framework and item selection. *Med Care*. 1992;30(6):473-483.
23. Ware J. SF-36 health survey update. *Spine*. 2000;25(24):3130-3139. <https://doi.org/10.1097/00007632-200012150-00008>
24. Ware J, Kosinski M, Keller SD. A 12-Item Short-Form Health Survey: construction of scales and preliminary tests of reliability and validity. *Med Care*. 1996;34(3):220-233. <https://doi.org/10.1097/00005650-199603000-00003>
25. Sintonen H, Pekurinen M. A generic 15 dimensional measure of health-related quality of life (15D). *The Journal of Society Medicine*;1989; 26: 85-96.
26. Sintonen H. *The 15D-measure of health-related quality of life. Reliability, validity and sensitivity of its health state descriptive system*. Melbourne: National Centre for Health Program Evaluation;1994.
27. Muth C, Harder S, Uhlmann L et al. Pilot study to test the feasibility of a trial design and complex intervention on PRIoritising MUltimedication in Multimorbidity in general practices (PRIMUMpilot). *BMJ Open*.2016;6(7):e011613. <https://doi.org/10.1136/bmjopen-2016-011613>
28. Muth C, Uhlmann L, Haefeli W et al. Effectiveness of a complex intervention on Prioritising Multimedication in Multimorbidity (PRIMUM) in primary care: results of a pragmatic cluster randomised controlled trial. *BMJ Open*. 2018;8(2):e017740. <https://doi.org/10.1136/bmjopen-2017-017740>
29. Taylor C, Byrd D, Krueger K. Improving primary care in rural Alabama with a pharmacy initiative. *Am J Health Syst Pharm*. 2003;60(11):1123-1129. <https://doi.org/10.1093/ajhp/60.11.1123>
30. Milos V, Rekman E, Bondesson Å et al. Improving the quality of pharmacotherapy in elderly primary care patients through medication reviews: a randomised controlled study. *Drugs Aging*. 2013;30(4):235-246. <https://doi.org/10.1007/s40266-013-0057-0>
31. Syafhan N, Al Azzam S, Williams S et al. General practitioner practice-based pharmacist input to medicines optimisation in the UK: pragmatic, multicenter, randomised, controlled trial. *J Pharm Policy Pract*. 2021;14(1):4. <https://doi.org/10.1186/s40545-020-00279-3>
32. Campins L, Serra-Prat M, Gózalo I et al. Randomized controlled trial of an intervention to improve drug appropriateness in community-dwelling polymedicated elderly people. *Fam Pract*. 2017;34(1):36-42. <https://doi.org/10.1093/fampra/cmw073>
